# EXTRA-ABDOMINAL DESMOID TUMOR: LOCAL RECURRENCE AND TREATMENT OPTIONS

**DOI:** 10.1590/1413-785220162403142182

**Published:** 2016

**Authors:** LUIZ EDUARDO MOREIRA TEIXEIRA, EUGÊNIO COSTA ARANTES, RAFAEL FREITAS VILLELA, CLAUDIO BELING GONÇALVES SOARES, ROBERTO BITARÃES DE CARVALHO COSTA, MARCO ANTÔNIO PERCOPE DE ANDRADE

**Affiliations:** 1. Universidade Federal de Minas Gerais, Department of Orthopedics and Traumatology, Belo Horizonte, MG, Brazil.

**Keywords:** Fibromatosis, aggressive, Medical oncology, Recurrence

## Abstract

**Objective:**

To evaluate the rate of local recurrence of extra-abdominal desmoid tumor and compare the outcomes of surgical treatment and conservative treatment.

**Methods:**

Twenty one patients (14 women and seven men), mean age 33.0±8.7 years old, with a diagnosis of desmoid tumor were evaluated. The mean follow-up period was 58.5±29.0 months. Fourteen cases involved the lower limbs, four cases involved the upper limbs, and three cases involved the trunk. The average tumor size was 12.7±7.5 cm. Of the 21 patients, 14 did not undergo previous treatment and seven patients relapsed before the initial evaluation. Surgical treatment was performed in 16 patients and conservative treatment was performed in five patients.

**Results:**

Recurrence occurred in seven patients (33%) and six of them relapsed within the first 18 months. No significant difference was observed between conservative and surgical treatment. However, a significant difference was observed among patients undergoing wide resection and who experienced improved local control.

**Conclusion:**

The recurrence rate of desmoid tumor was 33.3%. There was no difference in recurrence between conservative and surgical treatment. In surgical treatment, wide margins showed better results for recurrence control. ***Level of Evidence III. Retrospective Observational Study.***

## INTRODUCTION

Extra-abdominal desmoid tumor (fibromatosis) is a benign tumor of fibroblastic origin with distinct degrees of local aggressiveness and unpredictable biological behavior ranging from indolent and self-limited lesions to infiltrating and rapidly growing lesions. Although these tumors do not metastasize, they often present frequent local recurrence and high morbidity.^1^

These lesions are rare, corresponding to less than 3% of all soft tissue tumors. Most tumors develop sporadically with higher incidence among female patients. Although it can be diagnosed at any age, they are most commonly diagnosed in patients between 15 and 60 years of age. Genetic, endocrine, and physical factors, such as pregnancy and trauma, play an important role in the etiology of the disease. These lesions can occur anywhere in the body but are most frequently found in the abdominal wall and soft tissues of the extremities, shoulders, neck, dorsum, thigh, and chest wall.^1^

The optimal treatment has still not been established, although surgery is the primary treatment option. The local recurrence rate is high and varies between 15% and 77%.^2^ ^,^ ^3^ Other treatment options include chemotherapy, hormonal therapy, and radiotherapy, used either individually or in combination.^1^ ^,^ ^4^ ^,^ ^5^

The aim of this study is to evaluate the rate of local recurrence of extra-abdominal desmoid tumor and compare the outcomes of surgical treatment with those of conservative treatment.

## PATIENTS AND METHODS

This retrospective, observational study evaluated a series of patients diagnosed with extra-abdominal desmoid tumor, confirmed by histological examination. The treatments were performed at the *Hospital das Clínicas* at UFMG and Hospital Madre Teresa, located in Belo Horizonte, state of Minas Gerais, Brazil, between January 2002 and January 2014. This study was approved by the research ethics committees of the afore mentioned hospitals and was registered in the Brazilian Research Platform under protocol CAAE: 22514813.5.0000.5127.

A total of 21 patients were evaluated, of which 14 (66.7%) were females and seven (33.3%) were males. The age of the patients varied between 18 and 49 years, with a mean age of 33.0±8.7 years. The average follow-up period was 58.5 ± 29.0 months. The minimum and maximum follow-up periods were 16 and 127 months, respectively. With respect to tumor location, 14 (66.7%) occurred in the lower limbs, four (19.0%) in the upper limbs, and three (14.3%) in the trunk. The most frequent specific location was the popliteal fossa, observed in four (19.0%) patients. The average tumor size was 12.7 ± 7.5 cm, varying between 4.5 cm and 36.4 cm. Epidemiological data are summarized in Table 1.

**Table 1:**
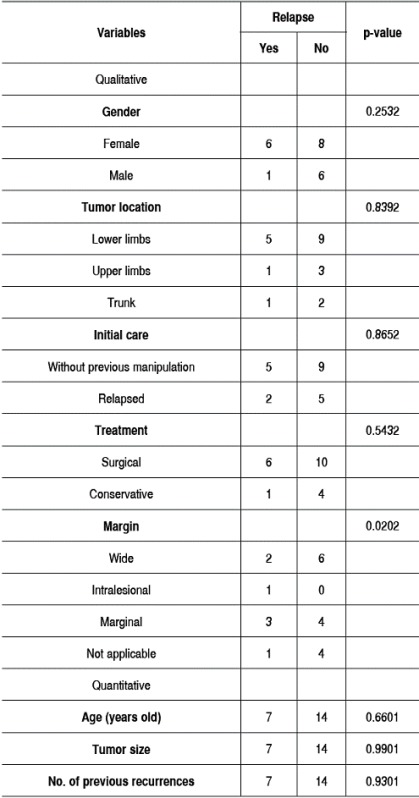
Comparison between recurrence period, gender, tumor location, initial care, treatment, margin size, age, tumor size, and number of previous recurrences.

1: Cox univariate model; 2: Log-rank test.

According to the initial treatment, 14 (66.7%) patients received no previous treatment and seven (33.3%) experienced local recurrence of previously manipulated tumor. Among the 21 patients, 16 (76.2%) underwent surgical treatment and five (23.8%) underwent conservative treatment. As for tumor margin in patients subjected to surgery, eight (50%) patients had wide margins, 7 (43.7%) were classified as marginal, and only one (6.3%) was intralesional. Adjuvant radiotherapy was performed in one (6.3%) patient.

Among the five patients subjected to conservative treatment, three underwent hormone therapy. The hormone of choice was tamoxifen for two (40.0%) patients and progesterone for one (20.0%) patient. The other two (40.0%) patients were treated with non-steroidal anti-inflammatory drugs and were under observation (expectant management).

Statistical analysis was performed using SPSS^W^ 17.0 software (Chicago, USA). The descriptive study was conducted using frequencies and percentages for the categorical variables, and the measures of central tendency (mean and median) and dispersion (standard deviation) were used for quantitative variables.

The response variable analyzed was local recurrence (surgery) or lesion progression (conservative treatment). This variable was compared to age, gender, type of treatment, prior manipulation, surgical margins, tumor location, tumor size, and number of previous recurrences.

For the comparative study, an univariate analysis was performed using the Kaplan-Meier method associated with the results of the log-rank test. A multivariate analysis was performed using the goodness-of-fit test for a Cox's regression model. The variables with p-values lower than 0.25 in the univariate analysis were used in the selection of covariates for adjustment of the final model. A value of p≤0.05 was considered statistically significant.

## RESULTS

**Figure 1:**
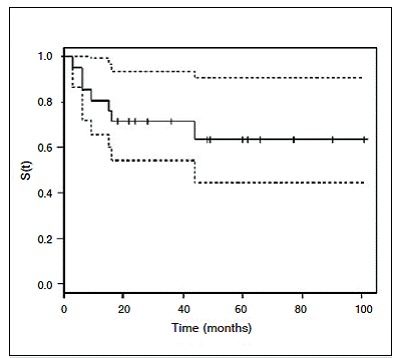
Recurrence-free survival curve (Kaplan-Meier) of patients subjected to treatment of extra-abdominal desmoid tumor.

Local recurrence/progression was observed in seven (33.3%) patients during follow-up and the average recurrence period was 14.1 ± 10.2 months. Six (85.7%) cases involving recurrence/progression occurred before 18 months. The survival curve is depicted in Figure 1. Among the relapsed patients, only one (14.3%) developed multiple recurrences.

The comparative analysis showed that gender (p = 0.253), age (p=0.660), tumor location (p=0.839), tumor size (p=0.990), and previous recurrence (p=0.930) did not significantly correlate with recurrence/progression after treatment. The data are summarized in Table 1.

**Figure 2:**
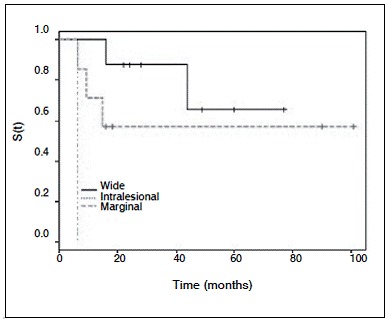
Recurrence-free survival (S) curve of patients subjected to surgical treatment of extra-abdominal desmoid tumor according to the surgical margin.

With regard to the disease treatment, the recurrence rate in the surgical treatment did not significantly differ from that in the conservative treatment. However, in patients subjected to surgery, the presence of wide margins was better and with a significant results (p=0.020) when compared with marginal and intralesional excision. (Figure 2) The relative risk of recurrence among patients with intralesional margins was 24.6 times higher than that of patients with wide margins (95% CI: 1.2-508.3).

## DISCUSSION

Desmoid tumor or aggressive fibromatosis was first described by MacFarlane in 1832. It is characterized by rare injuries with an incidence of 2-4 cases per 1.000,000 and it is predominant among young adults and females.^6^ Its etiology remains unknown but is believed to be a monoclonal fibroblast proliferation associated with increased production of ß-catenin protein, which stimulates fibroblast activity.^7^ It is typically characterized by firm and painless lesions, which harden and adhere to surrounding tissues. The growth is insidious but during its progression, it may cause significant functional limitation.

In the present study, we evaluated a series of patients with the epidemiological profile observed in most studies, with a mean age of 33.0 years and predominantly females. In contrast to other authors, who observed a higher frequency of lesions on the trunk and on the pectoral and pelvic girdle, we found greater involvement of the lower limbs.^8^ ^,^ ^9^

The tomor's biological behavior remains undefined and variable. Some lesions are aggressive, with multiple local recurrences and resistance to treatment. Tumors located in the head, neck, and chest can cause death by direct compression of vital structures. However, cases of spontaneous regression or regression after biopsy have been described.^10^ ^,^ ^11^

Frequent recurrence of these lesions has been widely reported, and the estimated recurrence rate varies between 15% and 75%. In our study, the recurrence rate was 33.3%, and in 85.7% of those cases, it occurred within 18 months after treatment.^9^ ^,^ ^12^

The treatment options included surgery, chemotherapy, hormonal therapy, and radiation therapy, either alone or in combination. Each of these treatments has demonstrated some degree of success. However, local recurrence remains as a problem.^1^ ^,^ ^9^

Surgical treatment with wide margins remains the method of choice for most patients with desmoid tumor.^2^ However, frequent recurrence and the functional and cosmetic sequelae of this procedure enables conservative treatment as a good option in cases of multiple recurrences, unresectable tumors, and in cases in which it is difficult to determine tumor location. We observed that wide margins are essential for the adequate control of fibromatosis, as previously reported by Duggal et al.^9^ Most authors acknowledge the importance of the surgical margin. However, Rock et al.^13^ did not find any benefit of extended margins for the control of desmoid tumors.

Among the conservative therapies, radiation therapy has been the most studied. Its application is usually described as a surgical adjuvant for large, unresectable tumors or in cases of contaminated margins. Jelinek et al.^14^ reported a local control rate of 53% in patients subjected to surgery and 81% in those patients subjected to surgery and radiotherapy.

The influence of hormones and the presence of estrogen receptors in these tumors has stimulated local control using hormone therapy, either as adjuvant or as main therapy.^1^ ^,^ ^15^ Virtually, all tumors express the nuclear estrogen receptor beta, but not all patients respond to anti-hormonal therapy.^16^

The best studied anti-estrogen is tamoxifen; however, most studies are based on a small sample size of patients, and no prospective studies using a sufficient number of patients are available to indicate the ideal dosage.^15^ ^,^ ^17^ Other anti-estrogen drugs used include progesterone, medroxyprogesterone, and testolactone.^15^

Recently, Briand et al.^18^ reported their experience with patient follow-up without treatment of desmoid tumor. Based on the findings by Gouin et al.^19^ and Barbier et al.,^20^ who indicated a high rate of spontaneous interruption of tumor growth, expectant management was adopted as the initial treatment, and the percentage of tumors without progression reached 90%.

Our study showed that the surgical and conservative treatment options are useful for local control of desmoid tumor. We believe that surgical internvetion is the best option for primary lesions, located where wide margins can be reached with low functional and cosmetic morbidity. Conservative treatment is an alternative for large lesions after multiple recurrences and in cases in which surgery will result in significant functional limitation.

## CONCLUSIONS

The recurrence rate of desmoid tumor observed in our sample was 33.3%. No difference in recurrence was observed between patients submitted to the surgical and conservative treatments. The wide margins showed better results for local control of the disease after the surgical treatment.
